# Psychosocial effects and quality of life after stoma surgery: systematic review and qualitative meta-synthesis

**DOI:** 10.1186/s40359-026-03993-w

**Published:** 2026-01-23

**Authors:** Seda Cansu Yeniğűn Akbulut, Ayşegül Ilgaz

**Affiliations:** 1 Kumluca Faculty of Health Sciences, Department of Surgical Disease Nursing, Akdeniz University, Antalya, Türkiye; 2 Department of Public Health Nursing, Akdeniz University Nursing Faculty, Antalya, Türkiye

**Keywords:** Stoma surgery, Surgical nursing, Psychological effect, Quality of life, Meta-synthesis study

## Abstract

**Background:**

Although stoma surgery is a life-saving intervention, it can lead to significant physical, emotional, and social changes that affect individuals’ psychosocial well-being and overall quality of life. This qualitative meta-synthesis aims to systematically investigate and synthesize the psychological and social impacts, as well as the quality-of-life experiences, of adult individuals living with a permanent stoma due to colorectal cancer.

**Methods:**

This qualitative meta-synthesis was conducted via systematic review and thematic synthesis methods, following the ENTREQ, PRISMA, and JBI guidelines. Five databases, EBSCO (MEDLINE), PubMed, CINAHL, Web of Science (WoS), and Scopus, were searched up to December 28, 2024. Studies focusing on the psychological, social, and quality-of-life experiences of adults living with a permanent stoma were included. The data were analysed via three-step thematic synthesis. Study quality was assessed via JBI tools. Two independent researchers conducted the selection, extraction, and synthesis processes to ensure reliability.

**Results:**

A total of 36 qualitative studies involving 1736 patients with stoma were included. Three main themes and six subthemes were identified: (1) Reshaping Daily Life in the Shadow of the Stoma, (2) From Inner Turmoil to Silent Resilience: Psychosocial and Emotional Effects After Stoma, and (3) Care and Support Dynamics in Stoma Adaptation. Patients experienced body image disturbance, emotional distress, social isolation, changes in daily routines, physical limitations, hygiene challenges, and stigma. Family, partner, peer, and professional support are key factors in coping and adaptation.

**Conclusion:**

Adults living with a permanent stoma reported experiencing physical limitations related to mobility, sleep, self-care, and sexual life, which were perceived to negatively influence their quality of life. Participants also described the need to reorganize daily routines, social roles, and work-related activities, and to develop new patterns of living within existing constraints. Overall, the findings suggest that living with a stoma is associated with adjustments in living arrangements, social roles, and bodily experiences, underscoring the importance of addressing the adaptation process through a holistic and person-centered approach.

**Trial registration:**

The study protocol was registered in the International Prospective Register of Systematic Reviews (PROSPERO) database (ID: CRD42025631485).

**Supplementary Information:**

The online version contains supplementary material available at 10.1186/s40359-026-03993-w.

## Background

According to the 2024 Global Cancer Observatory, colorectal cancer ranks as the third most common type of cancer after breast, prostate and lung cancer [1]. Early-stage colorectal cancer constitutes the primary form of this disease [2]. When anal sphincter-preserving surgery is inadequate, abdominoperineal resection—which typically requires permanent colostomy—is often the preferred approach [3, 4]. Ostomy surgeries may be performed due to cancer, inflammatory bowel disease, or pathologies such as diverticulitis. In these cases, an opening is created in the abdominal wall to divert the intestinal contents outside the body. Ostomy is a surgical procedure that allows urine or stool to exit the body through a surgically created opening called a stoma [5].

In the United States, approximately 725,000 to 1,000,000 individuals live with an ostomy or continent diversion, and nearly 100,000 ostomy-related surgical procedures are performed [6]. Despite being a life-saving intervention in many cases, stoma surgery adversely affects patients both physiologically and psychosocially, making it harder for them to adapt and lowering their quality of life [7, 8]. The fact that waste exits the body through an artificial opening rather than the natural route can provoke feelings of shame, alienation, loss of control, worthlessness, anger, and intense anxiety [9]. These emotions hinder participation in social life and undermine individuals’ confidence in being present in society, travelling, continuing to work, and fulfilling family roles [10].

Post stoma surgery, individuals undergo profound changes in body image and identity perception [11, 12]. A study by Stott et al. (2025) examined a group of community-dwelling individuals with ostomies, aiming to define their nine-month post-surgical adjustment process and to evaluate outcomes on the basis of personal and clinical characteristics [13]. This revealed that adaptation was not limited to the initial postoperative months but rather evolved over time across nine months [13]. Some individuals struggle to accept living with a stoma even years later and report difficulties in daily activities and a need for psychological support [14, 15]. Topics such as sexuality and intimacy are among the most challenging areas, and the lack of access to professional support in these areas further complicates the situation [16, 17].

Patients who undergo ostomy after colorectal cancer may experience psychosocial issues such as depression, anxiety, loss of self-esteem, social isolation, and shame. Additionally, these individuals may face limitations in daily activities, difficulties in work and social life, and changes in sleep and eating patterns, resulting in reduced quality of life. Previous meta-synthesis and meta-ethnography studies have focused on experiences related to living with a stoma, including general life experiences [17], impacts on sexual life [18], and overall quality of life [19], in individuals with stomas or diagnosed with colorectal cancer but have not directly examined the psychosocial and quality-of-life impacts of stomas. However, a review of the current literature revealed that, to our knowledge, there is no qualitative meta-synthesis that comprehensively examines the psychosocial experiences and quality of life of patients with ostomies after colorectal cancer. While a few primary qualitative studies have explored aspects such as body image, stigma, emotional distress, and social isolation, the findings remain fragmented and lack a unifying interpretation [10, 11, 20, 21]. Specifically, there is a significant lack of synthesized evidence regarding how patients reconstruct their identities after stoma surgery, how they adapt to changes in intimacy, how they navigate daily routines, and how they interact with social support systems. Therefore, this study highlights the need for a comprehensive synthesis that can offer a deeper understanding that can guide healthcare professionals in developing patient-centred care and support strategies. In this context, the aim of the meta-synthesis is to analyse, synthesize, and interpret the qualitative literature to provide an integrated understanding of the psychosocial challenges and quality-of-life experiences of colorectal cancer patients living with stomas.

### Research question

The research question was established on the basis of the PICOS framework.

- What are the psychological and health-related quality-of-life experiences of adult patients living with a permanent stoma after surgery, as explored through qualitative studies?

## Methods

### Study design

This study was conducted via a systematic review and qualitative meta-synthesis design, and the study protocol was registered in the International Prospective Register of Systematic Reviews (PROSPERO) database (ID: CRD42025631485). The meta-synthesis was carried out and reported in accordance with the Enhancing Transparency in Reporting the Synthesis of Qualitative Research (ENTREQ) statement guidelines [22].

Qualitative research provides in-depth insights into participants’ experiences. A qualitative meta-synthesis integrates findings from healthcare contexts to develop a theoretical and conceptual understanding for future health interventions [23]. This meta-synthesis was conducted following the Preferred Reporting Items for Systematic Reviews and Meta-Analyses (PRISMA) guidelines [24].

### Search strategy

For the search, five electronic databases were used: EBSCO (MEDLINE), PubMed, CINAHL, Web of Science (WoS), and Scopus. The MEDLINE database was researched via the EBSCOhost platform, while PubMed was searched separately to capture additional records not indexed in MEDLINE. Duplicate records retrieved across databases were identified and removed prior to screening. The keywords used in the database search were as follows: (stoma OR ostomy OR colostomy OR ileostomy) AND (psychological OR social OR psychosocial) AND (quality of life) AND (qualitative research OR qualitative study OR qualitative methods OR interview OR ethnographic OR phenomenological). The database searches included English-language articles from inception through December 28, 2024, without any restrictions on publication year (Supplementary File 1).

### Inclusion and exclusion criteria

The inclusion and exclusion criteria were developed on the basis of the PICoS framework (population, interest, context, and study design) recommended by the Joanna Briggs Institute (JBI) Centre for Evidence-Based Health Care in Australia [25]. The inclusion criteria were as follows: (1) population: patients with a permanent stoma due to colorectal cancer; (2) interest: patients’ experiences of psychological and social problems and quality of life; (3) context: experiences encountered in the clinical and social lives of patients with permanent colostomies; and (4) study design: qualitative studies and the qualitative components of mixed-method studies.

The exclusion criteria were as follows: (1) publication languages other than English; (2) articles with unavailable full texts or incomplete data; (3) gray literature; and (4) protocols and conference abstracts. The gray literature poses the danger of decreasing the comparability of results and the transparency/reproducibility of the synthesis because it varies greatly in terms of peer review, methodological depth, and reporting criteria. We restricted our search to full-text, peer-reviewed papers that offer adequate methodological information to obtain a more consistent quality rating in our meta-synthesis. Additionally, reviewing papers published in languages other than English through translation may increase the risk of conceptual shifts and methodological misinterpretations because the team doing this meta-synthesis did not include multilingual researchers. Therefore, articles written in languages other than English were removed to maintain the consistency of the assessment process and the validity of the results.

### Study selection and data extraction

Dual screening by two independent researchers was used to ensure methodical and transparent selection of research after the remaining references were moved to the Rayyan program. In the first stage, titles and abstracts were examined according to the inclusion and exclusion criteria determined to exclude articles irrelevant to the research topic. Second, the same researchers independently analysed the full texts of the papers to reduce the possibility of omission. Finally, research that satisfied all the inclusion requirements was chosen for meta-synthesis. If the researchers disagreed during this procedure, they debated and worked through the problem until they came to a consensus. Moreover, the two researchers’ consistency was assessed via Cohen’s kappa coefficient, which was 0.912, suggesting a high degree of agreement [26]. The integrity and dependability of the study selection procedure were greatly enhanced by the authors’ cooperation and consensus-building.

The relevant data were systematically recorded in a Microsoft Excel spreadsheet as follows: (i) general study and sampling characteristics, (ii) methods used, (iii) participants’ statements, and (iv) identified themes and findings. For mixed-methods studies, only qualitative components, such as interview findings, thematic descriptions, and participant quotations, were extracted and included in the synthesis. Quantitative findings were excluded from the analysis. All qualitative data, whether from purely qualitative or mixed-methods studies, were integrated via the same coding and synthesis procedure to ensure consistency.

### Data synthesis

This study used Thomas and Harden’s (2008) theme synthesis approach, which is based on a critical realism perspective [27]. Data synthesis was structured in a transparent, traceable, and reproducible manner in accordance with the JBI Evidence Synthesis Guidelines [22]. The findings sections and participant citations of the included studies were read multiple times to determine the units of meaning within the parameters of the analysis. One researcher (SCYA) coded the texts line by line, and another researcher (AI) independently reviewed the codes, assessing their applicability, scope, and consistency of code definitions. The coding system was created inductively; when new codes appeared, related codes were merged, their connections to themes became clear, and the codebook was updated. Any inconsistencies that arose among evaluators regarding code definitions, scopes, or thematic placement were resolved through iterative discussions, referring back to the original text until a complete consensus was reached.

Three steps were used to complete the thematic synthesis: (1) line-by-line coding was used to systematically label all the findings; (2) similar codes were grouped to create descriptive themes; and (3) relationships between the descriptive themes were established to develop analytical themes that went beyond the primary studies. The first step involved going line by line through each study’s findings text and participant citations to locate units of meaning. These units were then labelled “explicit codes” with terms that were as close to the text as possible. The code book included code definitions that were backed up by brief notes. In the second step, the codes were classified according to content similarity to identify the boundaries of the themes. In the third step, higher-level interpretive themes were generated by discussing interterm patterns and possible explanatory mechanisms. Excel was used for data organization, MAXQDA was used for coding and theme management, and disagreements were handled by consensus after consulting the text. To improve process traceability, Mendeley was utilized to standardize resource management, full-text editing, and citation/bibliography production procedures.

### Methodological quality assessment and appraisal

**Fig. 1:**
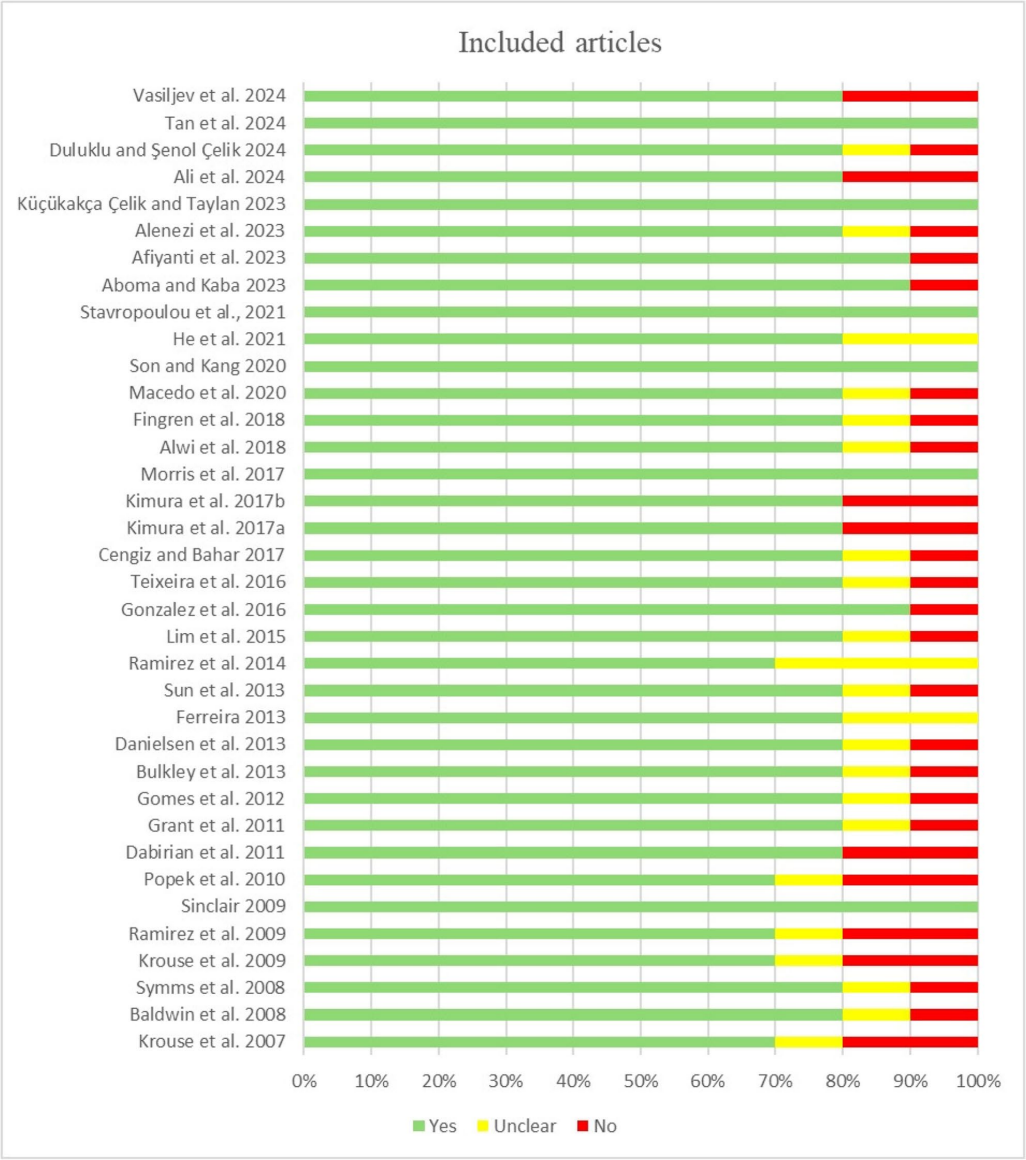
Quality assessment of included articles

After deleting 108 duplicates, 251 studies were selected for further review. Two pairs of reviewers examined the titles, abstracts, and full texts of these studies. The methodological quality of the included studies was independently evaluated by two trained, evidence-based researchers using the Joanna Briggs Institute (JBI) Critical Appraisal Checklist for Qualitative Research (2024 edition) [22]. The appraisal was based on 10 criteria, with each item rated as ‘yes,’ ‘no,’ ‘unclear,’ or ‘not applicable.’ The meta-synthesis included articles that were deemed to be of high quality or methodologically acceptable, with at least 60% of the evaluators responding “yes.” The risk of bias is shown as a percentage on the basis of how well each included study met the criterion (Fig. 1). The two researchers negotiated or had discussions to settle any disputes that arose throughout the review process.

### Confidence in the findings of synthesis

 The JBI-QARI was used to evaluate the methodological quality of the included studies, and the JBI Evidence Synthesis Guidelines recommended that the ConQual approach was used to rigorously analyse the reliability of the results synthesized by the meta-synthesis [28]. The JBI-QARI was used in this framework to assess the reliability of the studies that were part of the meta-synthesis; reliability was classified as high (4–5 items), moderate (2–3 items), or low (0–1 item) on the basis of items Q2, Q3, Q4, Q6, and Q7 on the checklist. Depending on how the findings related to the data, credibility was categorized as definite (clearly supported by data), doubtful (open to interpretation), or unsupported (lacking empirical support). Finally, the ConQual scores are classified as low, medium, or high (Table 1).

**Table 1:** An evaluation of the included studies’ quality (*n* = 36)

	Q1	Q2	Q3	Q4	Q5	Q6	Q7	Q8	Q9	Q10	Score*	Rating**
Tan et al. 2024 [10]	Y	Y	Y	Y	Y	Y	Y	Y	Y	Y	10	High
Ali et al. 2024 [29]	Y	Y	Y	Y	Y	N	N	Y	Y	Y	8	Moderate
Duluklu and Şenol Çelik 2024 [8]	Y	Y	Y	Y	Y	N	N	Y	Y	Y	8	Moderate
Küçükakça Çelik and Taylan 2023 [30]	Y	Y	Y	Y	Y	Y	Y	Y	Y	Y	10	High
Alenezi et al. 2023 [31]	Y	Y	Y	Y	Y	N	N	Y	Y	Y	8	Moderate
Afiyanti et al. 2023 [32]	Y	Y	Y	Y	Y	Y	N	Y	Y	Y	9	High
Aboma and Kaba 2023 [21]	Y	Y	Y	Y	Y	N	Y	Y	Y	Y	9	High
He et al. 2021 [20]	Y	Y	Y	Y	Y	N	N	Y	Y	Y	8	Moderate
Son and Kang 2020 [12]	Y	Y	Y	Y	Y	Y	Y	Y	Y	Y	10	High
Macedo et al. 2020 [33]	Y	Y	Y	Y	Y	N	N	Y	Y	Y	8	Moderate
Fingren et al. 2018 [34]	Y	Y	Y	Y	Y	N	N	Y	Y	Y	8	Moderate
Alwi et al. 2018 [35]	Y	Y	Y	Y	Y	N	N	Y	Y	Y	8	Moderate
Morris & Leach 2017 [14]	Y	Y	Y	Y	Y	Y	Y	Y	Y	Y	10	High
Kimura et al. 2017a [36]	Y	Y	Y	Y	Y	N	N	Y	Y	Y	8	Moderate
Kimura 2017b [37]	Y	Y	Y	Y	Y	N	N	Y	Y	Y	8	Moderate
Cengiz and Bahar 2017 [38]	Y	Y	Y	Y	Y	N	N	Y	Y	Y	8	Moderate
Teixeira et al. 2016 [11]	Y	Y	Y	Y	Y	N	N	Y	Y	Y	8	Moderate
Gonzalez et al. 2016 [39]	Y	Y	Y	Y	Y	N	Y	Y	Y	Y	9	High
Lim et al. 2015 [40]	Y	Y	Y	Y	Y	N	N	Y	Y	Y	8	Moderate
Ramirez et al. 2014 [41]	Y	Y	Y	Y	Y	U	U	Y	U	Y	7	Moderate
Sun et al. 2013 [42]	Y	Y	Y	Y	Y	N	N	Y	Y	Y	8	Moderate
Ferreira 2013 [43]	Y	Y	Y	Y	Y	U	U	Y	Y	Y	8	Moderate
Danielsen et al. 2013 [44]	Y	Y	Y	Y	Y	N	N	Y	Y	Y	8	Moderate
Bulkley et al. 2013 [45]	Y	Y	Y	Y	Y	N	N	Y	Y	Y	8	Moderate
Gomes et al. 2012 [46]	Y	Y	Y	Y	Y	N	N	Y	Y	Y	8	Moderate
Grant et al. 2011 [47]	Y	Y	Y	Y	Y	N	N	Y	Y	Y	8	Moderate
Dabirian et al. 2011 [48]	Y	Y	Y	Y	Y	N	N	Y	Y	Y	8	Moderate
Popek et al. 2010 [49]	Y	Y	Y	Y	Y	N	N	Y	N	Y	7	Moderate
Sinclair 2009 [50]	Y	Y	Y	Y	Y	Y	Y	Y	Y	Y	10	High
Ramirez et al. 2009 [51]	Y	Y	Y	Y	Y	N	N	Y	N	Y	7	Moderate
Krouse et al. 2009 [52]	Y	Y	Y	Y	Y	N	N	Y	N	Y	7	Moderate
Symms et al. 2008 [53]	Y	Y	Y	Y	Y	N	N	Y	Y	Y	8	Moderate
Baldwin et al. 2008 [54]	Y	Y	Y	Y	Y	N	N	Y	Y	Y	8	**Moderate**
Krouse et al. 2007 [55]	Y	Y	Y	Y	Y	N	N	Y	N	Y	7	**Moderate**
Vasiljev et al. 2024 [56]	Y	Y	Y	Y	Y	N	N	Y	Y	Y	8	**Moderate**
Stavropoulou et al. 2021 [57]	Y	Y	Y	Y	Y	Y	Y	Y	Y	Y	10	**High**

*Y, yes; N, no, U, unclear. JBI Critical Appraisal Tool score

**JBI ConQual score (Q2, Q3, Q4, Q6, Q7) Q1. Is there congruity between the stated philosophical perspective and the research methodology? Q2. Is there congruity between the research methodology and the research question or objectives? Q3. Is there congruity between the research methodology and the methods used to collect data? Q4. Is there congruity between the research methodology and the representation and analysis of data? Q5. Is there congruity between the research methodology and the interpretation of results? Q6. Is there a statement locating the researcher culturally or theoretically? Q7. Is the influence of the researcher on the research, and vice-versa, addressed? Q8. Are participants, and their voices, adequately represented? Q9. Is the research ethical according to current criteria or, for recent studies, and is there evidence of ethical approval by an appropriate body? Q10. Do the conclusions drawn in the research report flow from the analysis, or interpretation, of the data?

### Trustworthiness

Study selection, data extraction, appraisal, and synthesis were collaboratively carried out by two researchers (SCYA and AI) who are proficient in qualitative research methods. Throughout the process, the researchers aimed to achieve consensus by holding regular online and face-to-face meetings. The themes generated during the preliminary analysis were reviewed by all the authors, and the final themes were refined on the basis of this collaborative evaluation. Both researchers (SCYA and AI) had clinical experience with patients who had undergone stoma surgery. To enhance transferability, the study prioritized direct participant statements and detailed quotations over author interpretations. The findings were constructed via rich participant excerpts, supported by contextual information from each study. The reliability level of the results was ascertained via the quality assessment results. The research design, participant characteristics, and study setting are all thoroughly explained; additionally, the studies where the themes surfaced are given in a methodical manner (Tables 2 and 3).

**Table 2:** Characteristics of studies

CODE	Author, year, country	Aim	Study design	Setting/Context	Participants and age	Time elapsed after surgery	Themes
1	**Aboma & Kaba** **(2023)** **Ethiopia**	The aim of this study is to explore the lived experiences of individual with colostomy.	A phenomenological study	Public hospitals	9 participants with colostomy Age: 27–55 years	At least 3 months	**Psychological effect** Disbelief Shame Sleeping pattern disorder Accepting to live with colostomy **Social life** Difficulty in daily activity/movement Difficulty in keeping sexual life **Physical activity** Isolation/Stigma Difficulties in religious practice Social support **Economy** Financial hardship
2	**Afiyanti et al.** **(2023)** **Indonesia**	The findings of this study are expected to enhance the development of collaborative healthcare interventions which can accelerate sexual, psychological, and social recovery after ostomy among Indonesian ostomates.	A phenomenological qualitative study	National Cancer Center Hospital	12 female and male ostomates Age: 31–58 years	5 months-3 years	Sexual Disruptions Revealing Strategies for Sexual Adjustment Support from the Marital Partner Limited Support from the Healthcare Professionals
3	**Alenezi et al.** **(2023)** **Saudi Arabia**	The aim of this study is to explore the health- related quality of life outcomes and ostomy- related obstacles among patients with ostomy in Saudi Arabia.	This mixed- methods study	Hospital-based and outpatient settings	12 patients with ostomy Age: 18–75 years	Less than 1 year: 33.7% More than 5 years: 26.4%	**Stoma reshapes religious practices**, Difficulty to feel clean for prayer Physical limitations Spiritual distress associated with stoma **Apprehension of living with a stoma** Living with stoma brings increased self- awareness Fear of leakage **Adaptation** Adapting to changes in diet fluids and elimination Adapting to life with a stoma
4	**Ali et al.** **(2024)** **Malaysia**	This study was aimed to explore the perspectives on the acceptance, impact, and QOL CRC patients who must live with a stoma.	A phenomenological study	Hospital	12 Patients Age: 22–71 years	Within a month period	**Prior Knowledge** Information From the Attending Physician Patient’s Understanding of Stoma Formation and Function **Acceptance** Individual Acceptance Family Members’ Acceptance **Adaptation** Self-Managing of Stoma Support of Family Members **Challenges** Complication from The Stoma Social Life Financial Burden
5	**Alwi et al.** **(2018)** **Indonesia**	The aim of study was to describe the experiences of patients with end-stoma regarding their quality of life.	Descriptive phenomenology study	Hospital	12 patients with end-stoma Age:-	-	Becoming limited in doing daily activity Having limitation during sexual and social intercourse Having various negative feelings after the existence of end-stoma Having financial difficulties Attempting to survive with stoma Experiencing changes in fulfilling rest and sleep, physic, and complication Having expectation that must be achieved after having stoma
6	**Baldwin et al.** **(2008)** **USA**	To examine spiritual quality of life (QOL) of veterans with intestinal ostomies.	Mixed-method cross-sectional	healthcare settings	30 veterans with intestinal stomies Mean age: 69 years	Mean: 14.4 ± 12.2 (Quality of life upper) Mean: 6.3 ± 7.2 ((Quality of life lower)	Inner peace Hopeful Reason to be alive Positive changes in life Spiritual activities Religious activities
7	**Bulkley et al.** **(2013)** **USA**	Spiritual well-being (SpWB) is integral to health-related quality of life. The challenges of colorectal cancer (CRC) and subsequent bodily changes can affect SpWB.	This analysis combines qualitative and quantitative methods to study	Integrated healthcare system	92 individuals with ostomy reporting spiritual well-being- related challenges Mean age: 72 years	≥ 5 years	**Positive** Empowerment through positive attitude I am fortunate Faith in healers Active, full life Strength through religious faith Appreciate life more. “2nd chance” Helping others helps me **Negative** Loss Struggling to cope Not feeling “normal” Feeling let down/betrayed by healers **Ambivalent** Continuing to cope and function despite challenges Learning acceptance Payoff (Ostomy is the price for survival) Recovery from shock and trauma
8	**Cengiz & Bahar** **(2017)** **Türkiye**	The aim of this study was to determine perceived barriers to adaptation to life with a fecal ostomy based on the Health Belief Model and to reveal home care needs related to these perceptions.	Phenomenological study	Stomatherapy outpatient clinic of a university hospital	12 individuals with fecal ostomy Mean age: 54.41 ± 19.14 years	2–3 months	**Restriction of daily life activities** Getting dressed Bathing Sleep Sexuality Physical activity Prayers Social life **Factors Affecting Adaption to Stoma** Insufficient self-care Inability to accept Social support Chemotherapy and its effects **Needs for Health Care Professionals** Knowledge needs Problems due to stoma **Emotional effects**
8	**Dabirian et al.** **(2011)** **Iran**	The aim of study a qualitative study to explore quality of life and its dimensions in ostomy patients referred to the Iranian Ostomy Association.	Qualitative study	Iranian Ostomy Association	14 patients with ostomy Age: 14–57 years	1–10 years	Physical problems Psychological problems Social and family relationships Economic challenges Nutritional issues Physical activity Travel Religious considerations Sexual functioning
10	**Danielsen et al.** **(2013)** **Denmark**	The aim of the study was to explore the impact of a permanent stoma on patients’ everyday lives and to gain further insight into their need for ostomy-related education.	A phenomenological hermeneutic study	Hospital surgical department;	15 persons with permanent ostomies Age: 66 (median), range: 44–82 years	3 months (median), range: 3–5 months	Being different and Training in living a life with a stoma
11	**Duluklu & Şenol Çelik** **(2024)** **Türkiye**	To identify the lived experiences, quality of life (QoL), and level of ostomy adjustment (OA) in patients after colorectal cancer with a permanent colostomy (PC).	Hermeneutic interpretive phenomenological design	University hospital setting	14 participants Mean age: 61.5 ± 10.0 years	Mean: 7.7 ± 5.0 years	**Acceptance** **Medical problems** Treatment-related problems Ostomy related problems **Emotional changes** Regret Fear **Physiologic experiences** Sound and odor Sexual intercourse Daily activities **Psychosocial experiences** Managing negative emotions Social life Worship **Economic experiences** Increased expenses Coping strategies Emphasis on spirituality Taking up a hobby **Worries** **Expectations** Society Social environment/family Health services
12	**Ferreira** **(2013)** **Uruguay**	The aim of this study was to understand the life change events of a group of patients with a colostomy, as well as explore their nursing care expectations.	Qualitative study	-	9 patients with a colostomy Age: 49–72 years	≥ 3 months	The power of adaptation (because-motives) The social and job environment (becausemotives) The nurse as a consultant for the transition (in-order-to motives) The desire for compassionate care (in-orderto motives) Building new horizons (in-order-to motives)
13	**Fingren et al.** **(2018)** **Sweden**	The aim of this study was to assess patients’ adjustment to life with an ostomy and their quality of life (QOL) one year after surgery, and to identify individuals with lower adjustment levels. Additionally, the study aimed to explore obstacles to achieving a good quality of life.	Content analysis method according to Graneheim and Lundman	University hospital setting	101 patients with ostomy Age: median :70; range 21–90 years	1 year	Ostomy-related concerns and impact on life Limitations in physical and social activities Negative impact on physical and mental health
14	**Gomes & Brandão** **(2012)** **Brazil**	To identify factors that affect and change the daily lives of individuals enrolled in an ostomy association of a county in the state of Goias	Qualitative study	Community-based and outpatient settings	12 patients with ostomy Age: 31 and 78 years	Over six months	Understanding the purpose of making the stoma Knowing the ostomy care based on individual needs Unveiling of behavioral changes in the ostomates’ daily life
15	**Grant et al.**, **(2011)** **USA**	To describe how gender shapes concerns and adaptations among long-term colorectal cancer survivors with ostomies.	Qualitative study	Integrated healthcare system	33 colorectal cancer survivors with ostomies Age: 12 ± 8 years (males) 76 ± 9 years (females)	> 5 years	**Physical Well Being** Diet Gas & odor Activities Sleep **Psychological Well-Being** Coping & adjustment Humor Self-sufficiency Self-acceptance Body image Depression Unpredictability **Social well-being** Clothing Sexuality Travel Social support Embarrassment related to employment Financial issues **Spiritual well-being** Resilience/inner strength Appreciation for life Gratitude
16	**Gonzalez et al.** **(2016)** **Sweden**	The aim of this study was to explore wellbeing and body image 3 years after abdominoperineal excision in a population-based cohort of patients.	Mix-method design	Home-based, self-administered questionnaire	320 patients Age: 65 (range 59–71)	≥ 3 years	Bodily Limitations Stoma-Related Problems Sexual Dysfunction Tiredness/Fatigue Other Diseases Mental Suffering Ashamed of the Body Distress Acceptance Unchanged Everyday Life Positive Attitude Gratitude For Life
17	**He et al.** **(2021)** **China**	The aim of this study was to explore the immediate postoperative experiences before discharge among patients with rectal cancer and a permanent colostomy in China.	Qualitative study	University-affiliated cancer center	18 patients with permanent colostomy after rectal cancer surgery Age: 28 and 77 years	7 to 15 days	**Psychological Reactions** Stoma self-acceptance Negative emotions Social isolation **Daily Life Concerns** Daily life Misunderstandings Sexual life compromise Work restrictions **Stoma Care Considerations** Strong stoma self-care willingness Decreased stoma self-care confidence Access to high-quality stoma care **Support from Others** Enterostomal nurses Family members Stoma friends
18	**Kimura et al.** **(2017a)** **Brazil**	The objective of this study was to analyze the perceptions of oncological ostomized male individuals regarding sexual relations as an important dimension of quality of life, treated at the Ambulatory Care Program for Ostomized Patients of the Health Secretariat of the Federal District, Brazil.	Mixed method study	Outpatient (Ambulatory Care Program)	56 oncological ostomized individuals Age: 56.42 ± 12.16 years	**-**	**Ostomy** **Self-Care** **Acceptance** **Self-concept** **Companionship**
19	**Kimura et al.** **(2017b)** **Brazil**	This study aimed to acknowledge the perception of quality of life and the interpretation of biopsychosocial reality of the ostomized person due to colorectal cancer in outpatient clinics of the Ambulatory Care Program for Ostomized Patients of the Health Department, Federal District (DF), Brazil.	Descriptive study with qualitative approach	Outpatient (Ambulatory Care Program)	120 patients Age: 58.72 ± 12.56 years	-	**Physical Well-being** Leakage, odor and gases Physical strength Complications of ostomy Sleep Family support Care process in health **Psychological Well-being** Self-concept Self-care Stigma Acceptance and adaptation Family support Acceptance and adaptation
20	**Krouse et al.**, **(2007)** **USA**	It is essential to better understand the areas in which interventions may help to minimize the negative consequences.	Mixed methods design	Medical Centers; outpatient and survey-based setting	163 patients with ostomy Age: 68.8 ± 12.4 (cases) and 67.5 ± 11.5 (controls)	11.2 ± 11.6 years (cases) and 4.1 ± 3.0 years(controls)	**Physical** Activity/exercise Pain/discomfort Skin irritation Sleep **Psychologic** Coping/acceptance Fear of other’s perception Fear of ostomy “accidents” **Social** Travel limitations Sexuality/relationships Embarrassment Work/financial problems **Spiritual** Meaning to life **Ostomy-specific** Daily care Equipment problems/solutions Clothing restrictions **Medical care issues** Comorbidities Complications
21	**Küçükakça Çelik & Taylan** **(2023)** **Türkiye**	This study aimed to evaluate the perspectives of patients who had spinal cord injuries and were wheel chair dependent on colostomy surgery, which is among the bowel movement methods.	Heidegger’s hermeneutical phenomenological approach	Community-based setting	9 wellchair-dependent patients with spinal cord injury and colostomy Age: 32–52 years	**-**	**Difficult Experiences** Undesirable symptoms A long and boring job Psychosocial difficulties **Coping with Difficulties** **Colostomy Awareness Experience** People like me can understand me I have been left alone Colostomy can offer me hope
22	**Lim et al.**, **(2015)** **Singapore**	The aim of this study was to investigate patients’ experiences of performing self-care of stomas in the initial postoperative period.	Descriptive qualitative approach	Tertiary hospital setting; colorectal surgical ward with interviews conducted one month postoperatively	12 outpatients with colostomy and ileostomy Age: 58.2 ± 9 years (range: 40–72)	1 month	**Process of Acceptance and Self-management of Stoma** Unexpectedness Unpreparedness For Stoma Self-Care After Discharge Coping Issues with a Stoma **Physical Limitations** Activities Of Daily Living Personal Hygiene Poor Sleep Quality **Psychological Reactions** Frustrations Helplessness Afraid Of Being A Burden to Family Other Negative Feelings **Social Support** **Need for Timely and Sufficient** **Stoma Preparation and Education** Insufficient Education Received Recommendations On Future Education
23	**Macedo et al.** **(2020)** **Brazil**	To understanding the perception of ostomized patients affected by colorectal neoplasms regarding their quality of life	Qualitative research study	Specialized outpatient care center for ostomized patients	15 patients with ostomy Age: 55 ± 8 years (range: 37–65)	6 months-10years	Quality of life: social, psychic, and spiritual influences Personal and environmental adaptations considering the new reality Complications of living with an ostomy
24	**Krouse et al.**, **(2009)** **USA**	The goal of this qualitative analysis was to understand better patients’ perspectives regarding their greatest challenge.	Mixed methods Design	Affairs Health Care System; outpatient setting with mailed surveys and open-ended responses	163 patients with ostomy Age: 68.8 ± 12.4	11.2 ± 11.6 years	Positive thinking Adjustment over time Opportunity for reanastomosis Humor Recognition of positive changes resulting from stoma Normalization of life Meaning of life
25	**Morris et al.** **(2017)** **UK**	The aim of this explorative study was to describe and enhance understanding of pre- and post-ileostomy experiences in patients with Chron’s Disease to help inform clinical practice.	A phenomenological qualitative approach	Community and home-based setting	10 patients with ileostomy due to Crohn’s disease Age: 52.2 (range 34–83)	3–36 years	Controlling experiences in relation to life pre -ileostomy new life post ileostomy for Chron’s Disease
26	**Popek et al.** **(2010)** **USA and Canada**	The aim of this study was to qualitatively explore the health-related quality of life (HRQOL) concerns of veterans living with colostomies, using focus group interviews structured around the City of Hope Quality of Life framework.	A qualitative mixed-methods design	Medical Centers; focus group discussions in hospital-based outpatient settings	16 male veterans with colostomy -	-	Knowledge of own condition Effective/ineffective solutions to ostomy care Positive thinking/acceptance Family/spousal relationships Sexual relationships Loss of control
27	**Ramirez et al.** **(2009)** **USA**	The aim of this study was to explore, from an anthropological and phenomenological perspective, the sexual experiences, challenges, and adaptation strategies of female colorectal cancer survivors living with permanent ostomies.	A qualitative, phenomenological research design	Community and home-based settings	30-female patients Age: 44–93 (average age 70)	≥ 5 years	No Long-Term Sexual Difficulties Long-Term Sexual Difficulties Life course, Age-Related Changes in Sexuality No Sexual Experience Post Surgery
28	**Ramirez et al.** **(2014)** **USA**	The aim of this study was to explore how women living with an ostomy after colorectal cancer experience disruptions to full-adult personhood and how they regain a sense of personhood through technical and narrative adaptation processes.	Qualitative study	Community and home-based settings	30 female patietns with ostomy Age: 44–93 (average age 70)	≥ 5 years	**Changed Fecal Habitus as Disruption to Personhood** Strategies for Personhood Realignment after Ostomy Surgery **Learning a New Fecal Habitus** Narrative Reappraisals **Youth**,** Sex**,** and Pollution: A Gendered Lifecourse Reappraisal** Refusing Assaults to Personhood-Culture and Metaphor
29	**Son & Kang** **(2020)** **Korea**	The purpose of this study is to explore the coping experiences of individuals with ostomies throughout their illness, applying the Corbin and Strauss Chronic Illness Trajectory Framework, using exploratory qualitative methods involving focus group interviews.	An exploratory qualitative design	Community-based setting	19 individuals living with an ostomy Age: 70.8 ± 7.1 years (range: 57–82	1–30 years	Struggling and Suffering Learning How to Live with the Ostomy Living with the Ostomy
30	**Sinclair** **(2009)** **Canada**	The purpose of this study is to understand the experiences of young adults living with a permanent ileostomy.	Narrative inquiry qualitative study	Community-based setting;	7 patients with permanent ileostomy Age: 24–40 (range)	10 weeks-3.5 years	Postoperative Pain Management Nursing Care Stoma Concerns Impact On Body Image The Need For Ostomy Information And Education
31	**Stavropoulou et al. (2021)** **Greece**	The aim of the present study was to investigate the lived experience of patients undergoing permanent colostomy	A qualitative research design	Community-based; participants’ homes	8 patients with permanent colostomy Age: 48–77 years	1–12 years	**Experiencing a traumatic event** Extreme Emotions **Living a new reality** Personal changes Social adjustments **Efforts to improve quality of life** Autonomy Support
32	**Sun et al.** **(2013)** **USA**	The purpose of this paper is to describe persistent ostomy-specific concerns and adaptations in long-term (> 5 years) colorectal cancer survivors with ostomies.	A qualitative mixed-methods design	ntegrated healthcare system settings	163 colorectal cancer survivors with ostomies Age: 63 to 76 years	> 5 years	Bathroom issues Clothing restrictions and adaptations Dietary adjustments Irrigation Gas and odor problems Nickname for ostomy Ostomy bag duration Ostomy complications Ostomy family knowledge and acceptance Ostomy solutions Seatbelt Ostomy daily self-care Ostomy equipment failure Clothing adaptations Complications Equipment issues Coping and adjustment Activity limitations and adaptions Seatbelt issues Stoma site and size Leakage Seal and adhesive issues Skin issues Odor issues Pouch size issues Enclosure clip issues
33	**Symms et al.** **(2008)** **USA**	The aim of this study was examined quantitative and qualitative data to examine sexual functioning, intimate relationships, and health-related quality of life (HR-QOL) among military veterans who are living with an intestinal stoma.	A mixed-methods design	Health Administration sites; hospital-based outpatient and survey setting	154 military veterans living with an intestinal stoma Age: 68.3 ± 11.7	**2 years**	**Greatest Challenge** Sexual health Intimate relationships
34	**Tan et al.** **(2024)** **China**	To explore the post-surgery lived experiences of patients with colorectal cancer with a permanent ostomy for informing initiatives to improve patient care and future quantitative research.	A descriptive qualitative phenomenological	Tertiary hospital setting	12 patients with colorectal cancer and permanent ostomy Age: 46–62 years (range)	6–13 months	**Adapt to a new normal** Disease and treatment as a new normal Reshaping of everyday routines Challenges in daily care Reconstruction of quality of life **Rebalancing of physical and psychological status and** **social life** Loss and recovery of bodily autonomy Balancing privacy and disclosure Transformations in interpersonal relationships **Need for professional and social support** Professional health information seeking and education Need for a social support system
35	**Teixeira et al.** **(2016)** **Brazil**	The aim of this study was to analyze the perception of people with stoma elimination of their inclusion in the working world.	Qualitative, descriptive and exploratory Research	University hospital general surgery outpatient clinic	7 individuals with stoma elimination Age: 38–47 (range)	1–7 years	Meaning of work for people with stoma elimination The dialectic of work issue to be ostomy
36	**Vasiljev et al.** **(2024)** **Croatia**	The aim of this study was to qualitatively analyze QoL in stoma patients and to gain an insight into patient experiences in different QoL areas, as well as to determine patient satisfaction with health education provided by hospital nurses	Qualitative descriptive study	Hospital and community setting	15 stoma patients (6 women, 9 men) Age:40–86 years	mean 7.7 years (range: 1–27)	Health education and knowledge Psychological adaptation and acceptance Body image and self-perception Physical functioning Social life and participation Family and social support Economic impact Nutrition and daily life adaptations

**Table 3:** Themes of meta-synthesis (*n* = 36)

Synthesis Findings	Categories	Aboma & Kaba (2023)	Ramirez et al. 2014	Dabirian et al. 2011	Ferreira 2013	Afiyanti et al. (2023)	Alenezi et al. (2023)	Ali et al. 2024	Alwi et al. (2018)	Baldwin et al. (2008)	Bulkley et al. (2013)	Cengiz & Bahar (2017)	Danielsen et al. (2013)	Duluklu & Şenol Çelik (2024)	Fingren et al. (2018)	Gomes & Brandão (2012)	He et al. (2021)	Kimura et al. 2017a)	Kimura et al. 2017b)
Reshaping Daily Life in the Shadow of the Stoma	Daily Struggles Under the Weight of Bodily Limitations	✔		✔	✔		✔	✔	✔			✔	✔	✔	✔	✔			✔
	A New Order, Broken Habits and Transformed Roles	✔		✔	✔	✔		✔					✔	✔	✔	✔		✔	✔
From Inner Turmoil to Silent Resilience: Psychosocial and Emotional Effects After	Distorted Self-Perception and Negative Emotional Experiences	✔		✔	✔	✔	✔	✔	✔			✔		✔	✔		✔	✔	✔
	Inner Empowerment and Hopeful Outlook Toward the Future				✔	✔		✔		✔	✔			✔				✔	✔
Care and Support Dynamics in Stoma Adaptation	Hygiene and Care Needs Related to the Stoma			✔				✔				✔				✔	✔	✔	✔
	Sources of Strength in Stoma Adaptation: Support from Family, Friends, and Healthcare Professionals			✔	✔	✔		✔				✔		✔			✔	✔	✔

Synthesis Findings	Categories	Küçükakça Çelik & Taylan (2023)	Grant et al., (2011)	Gonzalez et al. 2016	Macedo et al. (2020)	Lim et al. (2015)	Krouse et al. (2007)	Krouse et al. (2009)	Son & Kang (2020)	Ramirez et al. (2009)	Tan et al. (2024)	Popek et al. (2010)	Symms et al. (2008)	Sun et al. (2013)	Teixeira et al. (2016)	Sinclair (2009)	Morris & Leach (2017)	Vasiljev et al. (2024)	Stavropoulou et al. (2021)
Reshaping Daily Life in the Shadow of the Stoma	Daily Struggles Under the Weight of Bodily Limitations	✔	✔	✔	✔	✔	✔	✔	✔		✔			✔		✔	✔	✔	✔
	A New Order, Broken Habits and Transformed Roles	✔	✔	✔	✔			✔	✔		✔			✔			✔	✔	✔
From Inner Turmoil to Silent Resilience: Psychosocial and Emotional Effects After	Distorted Self-Perception and Negative Emotional Experiences	✔	✔	✔	✔	✔	✔		✔	✔	✔		✔		✔	✔		✔	✔
	Inner Empowerment and Hopeful Outlook Toward the Future	✔	✔	✔				✔	✔		✔	✔		✔			✔	✔	✔
Care and Support Dynamics in Stoma Adaptation	Hygiene and Care Needs Related to the Stoma					✔	✔					✔		✔		✔	✔	✔	✔
	Sources of Strength in Stoma Adaptation: Support from Family, Friends, and Healthcare Professionals	✔	✔			✔	✔				✔	✔			✔		✔	✔	✔

### Ethical consideration

Ethical approval was not required because all included studies had been previously approved by their respective institutional review boards.

## Results

### Search findings

**Fig. 2:**
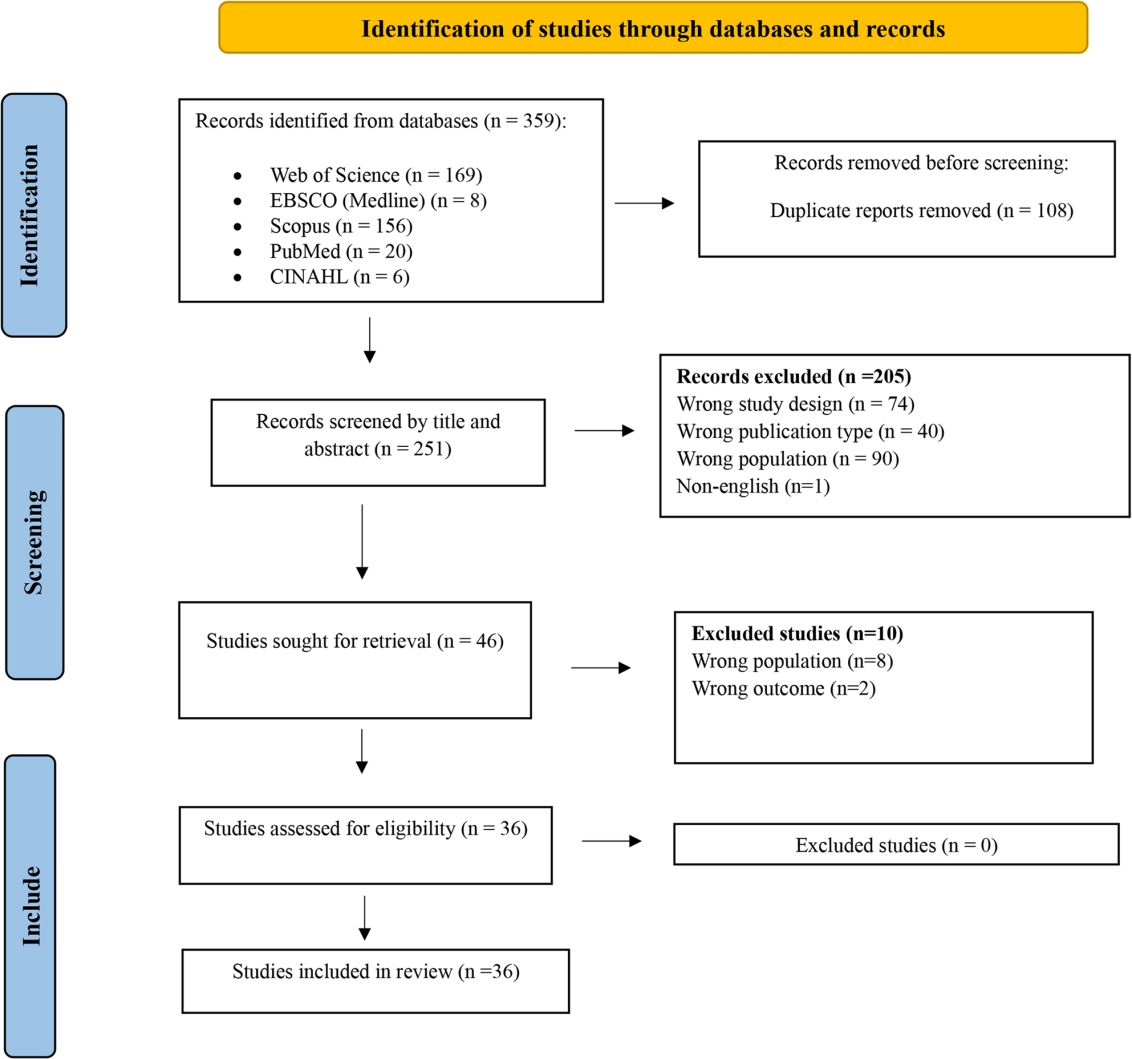
PRISMA Flow Diagram

As presented in Fig. 2, after removing duplicate records and studies excluded on the basis of title and abstract screening, 46 studies were selected for full-text assessment, and ultimately, 36 studies were included in this meta-synthesis (Fig. 2; Table 2).

### Characteristics and quality appraisal of the included studies

The 36 studies included in this review were conducted or published between 2007 and 2024 and covered a wide range of geographical areas. Asia (*n* = 7), Europe (*n* = 6), the Middle East and Africa (*n* = 6), South America (*n* = 6), and North America (*n* = 11; including the US and Canada) were the locations of the studies. Most studies used a qualitative research approach; phenomenological designs (*n* = 8) and other qualitative designs (*n* = 8) were the most reported designs; mixed methods studies also played a significant role (*n* = 9). Additionally, there were three hermeneutic phenomenological study designs, three descriptive qualitative research designs, two exploratory qualitative research designs, and one narrative research design.

The age range of the participants in the studies was 18–93 years, and most of the samples were from middle-aged to older age groups [12, 31, 34, 45, 51]. There have been reports of a wide range of postoperative times, such as the incredibly early time before discharge (7–15 days), early recovery times of 1–3 months, midterm times of 6–13 months/1 year, and long-term experiences spanning 1–30 years and 3–36 years [10, 12, 14, 20, 40, 44]. Community/home-based settings (*n* = 10), hospital/university hospitals and surgical wards (*n* = 8), stoma therapy-specialized ostomy outpatient units and other outpatient settings (*n* = 8) were the main settings in which the included studies were carried out. Studies with undefined contexts or generic statements (*n* = 2), cancer centre’s (*n* = 2), and institution-based structures (*n* = 6), such as integrated health systems/health administration and ostomy associations, were included.

A total of 1736 people with stomas participated in the studies when the “number of participants” from the 36 studies listed in Table 2 was considered (study samples ranged from 7 to 320 people). Some studies have concentrated on male-only samples, such as male veterans [49], women with ostomies following colorectal cancer [41, 51], or wheelchair-dependent people with colostomies due to spinal cord injury [30]. These studies address the psychological (acceptance-adaptation, self-image, anxiety), social (relationships, privacy, work/economy), physical (leakage/odour, challenges, limitations in daily life), and spiritual/religious elements of having a stoma (Table 2).

### Thematic synthesis

**Fig. 3:**
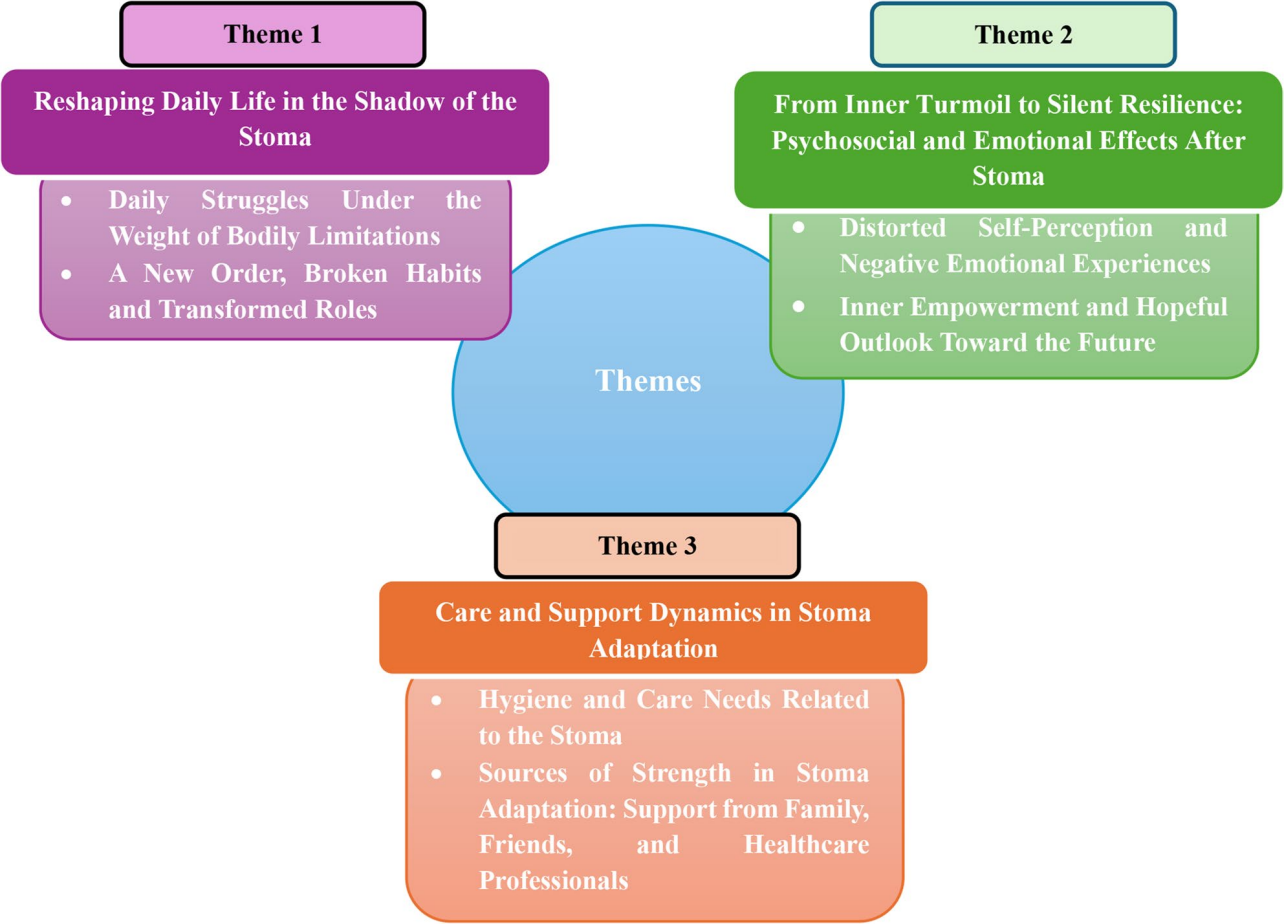
Main themes and subthemes

In this qualitative meta-synthesis, the psychological, social, and quality-of-life experiences of adults living with a stoma were categorized through thematic analysis into three analytical (main) themes and six descriptive (sub)themes. These themes are supported by direct participant quotations extracted from the studies and are presented in detail. Supporting excerpts and related findings from all included studies are shown in detail according to the themes (Fig. 3). These results have been condensed into six groups, which are shown in Table 3, on the basis of the similarity of meanings.

### Theme 1. Reshaping daily life in the shadow of the stoma

This theme describes the challenges individuals with a stoma face in daily activities such as physical activities, self-care, sleep, and sexual life, as well as the processes of restructuring these functions. This process is examined through two subthemes: “Daily Struggles Under the Weight of Bodily Limitations” and “A New Order, Broken Habits and Transformed Roles.”

### Daily struggles under the weights of bodily limitations

This subtheme focuses on the physical difficulties faced by individuals with a stoma in their daily lives following stoma surgery. Studies have shown that structural changes caused by stomas lead to problems such as restricted body movement, fatigue, sleep disturbances, and significant limitations in dressing, bathing, and physical activity [10, 21, 34, 35, 38, 40, 47, 48, 55]. Inability to exercise, constant pain, and persistent fatigue are also commonly described problems [35, 55, 56].

Sleep problems are another significant factor affecting the quality of life of individuals with stomas. Some studies have indicated that after stoma surgery, individuals experience difficulty falling asleep, frequent awakening, reduced sleep duration, and a lack of rest due to fragmented sleep [15, 21, 35, 38, 40, 47, 55, 56].

In addition, the negative impact of stomas on individuals’ sexual lives was highlighted. Factors such as sexual dysfunction, body image distortion, and fear of rejection by partners have caused some individuals to avoid sexual activity and create emotional distance in their relationships [20, 32, 38, 51, 53].

“It was tough to lie down and stand up after the colostomy surgery” (Aboma & Kaba, 2023, p.18, male, age: 37).

“This is really troublesome. It is troublesome when sleeping as well. Sometimes, at night, while sleeping, I have to wake up 3–4 times. I don’t get sufficient sleep. At night, you have to wash it clean, then sleep, then it will still come out, and get full after a while” (Lim et al., 2015, p.189).

There was no erection. My wife is not interested in sexual relations. Individuals with colostomies also reported that they do not have any sexual relationships because they are concerned that their partners may harm their colostomy. “Our sex life has come to an end”. In truth, there is no intimacy. There will be no intimacy since it may smell or produce gas. My wife is also affected. Our intercourse frequency is quite low” (Aboma & Kaba, 2023, p.17, male, age: 31).

“I used to run ten, twelve miles a day. Now I just walk. It does not bother me with walking too.

much. Running seems to just agitate my system to the point where anything in there, it works it out” (Grant et al., 2011, p.16).

### A new Order, broken habits and transformed roles

This subtheme encompasses the forced changes that individuals with a stoma face in their daily routines, social habits, and life roles. Studies have shown that individuals must restructure their eating habits, sleep patterns, travel planning, and how they spend time outside the home [10, 15, 21, 40, 42, 52]. Furthermore, the preservation of privacy and perceptions of sexuality were redefined within this new lifestyle [32, 53].

Many individuals expressed that they had to adjust their working arrangements or even quit their jobs because of the physical challenges encountered in the workplace [11, 42]. Factors such as social isolation, fear of travel, and difficulty finding toilets in unfamiliar places have led to a more controlled and restricted lifestyle [14, 20, 34, 39, 47, 55], with additional financial burdens further reshaping daily routines and limiting participation in community life [29].

“It means to me that, physically, I’m immobilized. In a way, I cannot do running; I cannot do swimming; I cannot do cycling, which is part of my daily routine. My work is also affected: I cannot move about that smoothly; I cannot climb I. My movement: I cannot squat down or walk faster. When I drive, I have to put the seat belt under the stoma. The seat belt is very close to the stoma, I cannot go over it, I have to go under it. It becomes a problem, and sometimes I never wear it [the seat belt]; so, I have to drive slower. Your mobility will slow down; your efficiency will drop. When you go out to the public place for dinner, such as restaurants, it is difficult to find a toilet with tap where you can do the draining. Furthermore, the bag gets filled up very frequently; so we have to avoid going for buffets or lunch appointments” (Lim et al., 2015, p.189).

“I’m in no rush to have it reversed because my quality of life has improved tenfold. I’ve retrained at college. I have a new profession; I work full time, so life isn’t too bad” (Morris & Leach 2017, p.37).

“I cannot wait to return to my activity. I have a great desire to return to work because I’m going to be busy doing anything that occupies the time and makes me feel useful” (Teixeira 2016, p.71).

“Unfortunately, I would rather stay at home, can’t cope with being as social as earlier …don’t have the strength to do what I want to affect by ageing..have less strength and stamina.” (González et al. 2016, p.1714).

### Theme 2. From inner turmoil to silent resilience: psychosocial and emotional effects after stoma

This theme encompasses the initial intensity of the psychological challenges experienced by individuals and the subsequent development of psychological resilience over time. To understand this process, two subthemes were identified. These are “Distorted Self-Perception and Negative Emotional Experiences” and “Inner Empowerment and Hopeful Outlook Toward the Future”.

### Distorted Self-Perception and negative emotional experiences

Studies have indicated that individuals experience not only physical but also profound psychosocial and emotional transformations following stoma surgery. During this internal journey, which begins post operation, individuals frequently go through stages of shock and denial [8, 10, 22, 25, 30–32, 45, 55], struggle and suffering [12], depression [14, 47, 50], stigma [21, 41], and intense emotional responses such as fear, loneliness, and anxiety [15, 20, 40, 53, 55].

Some studies noted that individuals experienced a disrupted self-image, a sense of losing bodily integrity, social withdrawal, and feelings of being “abnormal,” all of which negatively affected their daily lives [14, 50]. Furthermore, individuals encounter not only physical but also mental and emotional stress during social activities and travel. The constant need to carry stoma supplies, the anxiety of locating suitable restrooms while travelling, and the potential for the pouch to leak or release gas were identified as factors that limited participation in social activities and compelled individuals to plan their travel in detail [42, 47]. In this context, the restriction of spontaneous movement in daily life and the constant need to be prepared for unforeseen situations were highlighted as ongoing sources of tension [8, 20, 42, 47, 55].

“That tragedy was too much for me to bear. When I opened my eyes, I couldn’t look at the colostomy site. I did not investigate what had happened. I did not even look at it.” (Aboma & Kaba, 2023, p.16).

“I am unable to join the community. This is my most significant issue. We used to meet on specific days, but I am no longer able to attend.” (Aboma & Kaba, 2023, p.18, male, age: 32).

Life without it (the stoma) was terrible. I was constantly looking for a toilet. My employer thought I was not interested in work; I nearly got sacked for having so much time off…I got truly low then (Morris & Leach, 2017, p.37).

Your spouse or one of the children always helps. Going to the toilet and cleaning afterward.

cannot be managed alone. You lose all your privacy; that’s, you get ashamed and sad…” (Küçükakça Çelik & Taylan, 2023, p.1985, Female, age:42).

I could endure any kind of physical suffering, but I dread the derision that I am an abnormal person and feel humiliated as a sinner until now (He et al., 2021, p.3).

### Inner empowerment and hopeful outlook toward the future

The reviewed studies reported that, despite the psychological difficulties experienced initially after stoma surgery, individuals gradually adapted to their new condition and reconnected with life. Some participants described the experience as a “second chance” and developed a deeper sense of gratitude toward life [8, 15, 42, 45, 47, 51, 55]. People began to see living with a stoma as a necessity and a price to be paid for survival, and this perspective helped them accept their condition and create a new way of life [40, 41, 50, 52].

Other studies have emphasized that self-acceptance and positive thinking enhance individuals’ psychological resilience; some even manage the process using humor and an optimistic outlook [8, 12, 42, 49].

“Another challenge was learning to accept the fact that I would have a colostomy the rest of my life. It’s not something I ever thought about, so it was a complete surprise to me” *(Bulkley* et al., *2013*,* p. 2515).*

“I sincerely feel that I am here for a reason and having an ostomy or any physical problems [is not] going to keep me from achieving that reason, whatever it may be” *(Krouse* et al., *2009*,* p.230*,* Male*,* age: 79).*

“I attend courses of jewellery, leather bag fabrication, and painting three days a week. I am going out and chatting with my friends and doing something. I am not dwelling on my thoughts.” *(Duluklu & Şenol Çelik*,* 2024*,* p. 316*,* female*,* age: 62)*.

I took a photo with my stoma and sent it to my microblog with the words “This is my poached egg that will accompany with me my whole life” *(He* et al., *2021*,* female – 42 years).*

### Theme 3. Care and support dynamics in stoma adaptation

This theme encompasses the experiences of individuals with a stoma in relation to care processes and support mechanisms. In addition to physical care needs following stoma surgery, individuals also encounter various challenges and opportunities in seeking social support. In the process of coping with a stoma, factors such as hygiene, the need for information, professional support, and support from family and the social environment were found to play important roles in the adaptation process. The subthemes identified within this context are “Hygiene and Care Needs Related to the Stoma” and “Sources of Strength in Stoma Adaptation: Support from Families, Friends, and Healthcare Professionals”.

### Hygiene and care needs related to the stoma

The studies reported that individuals who underwent stoma surgery faced numerous physical and psychosocial challenges in hygiene and care processes. In particular, issues such as pouch leakage, gas, unpleasant odor, improper pouch placement, skin irritation, and itching have been highlighted as complications that adversely affect daily quality of life [20, 29, 35, 42, 44, 47, 49, 55].

Some participants noted that a lack of knowledge about stoma care led them to perform incorrect practices and that they did not receive sufficient professional support during this period, which in turn fostered feelings of loneliness and helplessness [8, 30, 40, 45]. The difficulties in maintaining hygiene are depicted as having not only physical but also psychological and social impacts [33, 51, 54]. Some people have even said that this illness makes it difficult for them to engage in spiritual or religious activities [15, 58].

“I have a serious prolapse that protrudes and becomes irritated. This condition also keeps me from swimming or using public showers” (Sun et al., 2013, p.18).

“I try everything at home for odor control, and it is just an odorous thing. There’s not a lot you can do about it” (Grant et al., 2011, p.18).

“My main problem is that adhesive on a bag is not good enough to last all day to support my active lifestyle” (Sun et al., 2013, p.19).

“The presence of ostomy affects the ablution, so I feel that I am not clean” (Alenezi et al., 2023, p. 3713).

*“I had a lot of irritation on my skin […].”* (Kimura et al. 2017b, Female, p.5).

### Sources of strength in stoma adaptation: support from the Family, Friends, and healthcare professionals

In the process of living with a stoma, individuals’ ability to cope with physical and psychosocial challenges was described to be largely influenced by environmental support mechanisms. Support from spouses, family members, friends, peers with similar experiences, and healthcare professionals plays a crucial role in helping individuals manage life with a stoma [8, 29, 30, 32, 42, 45].

Spousal or partner support was especially critical for rebuilding one’s body image, preserving intimacy, and regaining a sense of confidence in one’s sexual life. Family support helps reduce feelings of loneliness, share the burden of care, and provide emotional security [20, 21, 43, 47, 49, 50]. Acceptance from the social environment helped reduce the fear of stigma and facilitated reintegration into social life. Interactions with others who had similar experiences were found to enhance individual adjustment through shared empathy, mutual support, and a sense of normalization [20, 51].

Healthcare professionals, particularly stoma care nurses, were highlighted as key figures in enhancing patients’ knowledge, improving care skills, and boosting self-confidence [40, 44]. Postdischarge support is also important; some individuals exchange information and receive emotional support via support groups and social media platforms, which helps make the experience of living with a stoma more meaningful [21, 36, 37, 42].

“My husband was also present. ‘Oh! You can do and learn,’ he said.” (Aboma & Kaba, 2023, p.19, female, age:47).

“My doctor said that this surgery would not be optional; even if it were not, the insurance would not cover it. I wish he would support me.” (Küçükakça Çelik & Taylan, 2023, p.1985, Male, age:48).

“I am too old to undergo colostomy surgery, but my sons persuaded me. I always obey my sons’ decisions” (He et al., 2021, p.4, Female, age:74).

“I volunteered at a hospital for 14 years to go and talk to the new ostomy patients and met with others at a different hospital in a support group. I found this activity very helpful” (Bulkley et al., 2013, p.2517).

“The best thing is that your hospital invited two stoma visitors to help me by taking them as examples. I had unbounded confidence and was not scared of the stoma any more” (He et al., 2021, p.4, Male, age: 70).

“My family gave me the courage to live with this pouch.” (Kimura et al. 2017a, p.202).

## Discussion

The synthesized findings of 36 qualitative studies with adults who have stomas have shed light on the psychological, social, and quality-of-life experiences of these patients. This study is distinctive because it comprehensively synthesizes fragmented qualitative findings into a holistic interpretation of the psychosocial experiences and quality of life (including identity reconstruction, adaptation to intimacy, daily routines, and interaction with social support) of individuals with colorectal cancer ostomies, going beyond the general experience, sexuality, or overall quality of life focus of previous meta-synthesis/metaethnography studies [17–19]. Additionally, this synthesis includes social contacts, psychological moods, and everyday activities in addition to physical factors, providing a thorough evaluation of the life experiences of people with stomas. Through findings classified under three main themes, this meta-synthesis ensures insight into how people with stomas adjust to this process, how their lives are transformed, and which support systems work best for them during this adaptation process. This study emphasizes the necessity of a biopsychosocial and holistic approach to care, taking into account the psychological, social, and environmental changes that people with stomas experience in addition to their physical alterations.

### Reshaping daily life in the shadow of the stoma

People who have stomas deal with a number of physical issues, including limited mobility, exhaustion, sleep issues, trouble taking care of themselves, and the inability to exercise on a daily basis following surgery. Furthermore, stoma surgery has a detrimental effect on sexual life, leading to distance in partner relationships, sexual dysfunctions, and reduced body perception. According to research in the literature, stoma surgery results in major health issues, such as reduced body mobility, persistent exhaustion, sleep disturbances, and problems with sexual function [57, 59–61]. A person’s privacy, self-care, and self-perception may all be directly impacted, which could lower their quality of life in addition to physical changes. Issues such as disturbed sleep cycles, nighttime awakenings from changing bags, and inadequate sleep also significantly impair people’s capacity to function in their daily lives [40, 47]. Additionally, people who experience sexual dysfunction isolate themselves from sexuality and from their spouses [20, 32]. People with stomas face physical and social challenges that significantly impair their quality of life by affecting not only their physical functioning but also their psychological integrity. Therefore, to increase the quality of life for people with stomas, comprehensive support services such as physical rehabilitation, sleep management, and sexual counselling should be created.

People’s lifestyles have had to adapt due to the physical constraints of a stoma. According to research, people with stomas must reorganize a variety of daily routines, including their eating habits, preferred modes of transportation, job styles, and social activities [5, 42]. Uncontrollable circumstances, such as the fear of not finding a bathroom or the worry that the bag will leak or fill up, lead people to abandon spontaneous living and choose a more regimented, solitary lifestyle [10, 42, 51]. People have changed their working conditions or withdrawn from their professional responsibilities as a result of the physical barriers imposed by stomas [33, 54]. These findings demonstrate that the stoma is an adaptive process that impacts many facets of life and changes people’s identities, bodily roles, and lifestyles. In light of these issues, health care providers should develop individualized treatment regimens and counselling programs that promote living skills for stoma patients.

### From inner turmoil to silent resilience: psychosocial and emotional effects after stoma

The intense emotional upheaval that people with stomas initially experience eventually becomes inner strength. People who have a stoma frequently struggle with emotions, including low self-esteem, humiliation, stigma, and loneliness; these can have detrimental effects, such as social disengagement, loss of one’s work, and persistent worry. However, many people eventually build psychological resilience by accepting their circumstances and reintegrating into society [20, 45, 47, 50, 52]. Coping mechanisms, including humour, constructive pursuits, and optimistic thinking, help people manage this process more healthily [8, 12]. Their quality of life improves as a result of their acceptance of their stoma and the coping mechanisms they employ during this phase [62]. People with stomas rebuild themselves during this period, going through a spiritual and psychological metamorphosis. Over time, people with stomas can become more resilient inside themselves and turn a horrific event into a worthwhile life experience. Given these findings, it is advised that psychosocial support programs be designed to enhance people’s processes of self-acceptance and prioritize interventions that increase resilience in these processes.

### Care and support dynamics in stoma adaptation

People have found post stoma hygiene and care procedures to be emotionally and technically taxing. Issues that immediately impact people’s quality of life include bag leaks, unpleasant odors, skin irritation, incompatibility of materials, and a lack of care instructions [20, 35, 42, 47]. These challenges encompass physical, emotional, and social sensations of insufficiency, shame and powerlessness. They might also have an impact on certain people’s spiritual pursuits [15, 30]. People engage in inappropriate behaviours and feel abandoned as a result of inadequate knowledge about stoma care and a lack of expert aid [8, 40]. People with stomas suffer from physical and mental distress as a result of stoma leakage, which has a major negative influence on their quality of life [63]. Patients complain about social issues and anxiety as a result of feeling ignorant about their ostomies [64]. Individuals’ physical comfort and psychological adjustment are severely harmed by technical flaws and ignorance in post stoma hygiene and care procedures. People with stomas should have access to structured follow-up programs that offer psychosocial support and thorough care education.

Environmental support systems are as crucial to stoma management as the ability to provide care. Research has shown that maintaining one’s sense of privacy, developing emotional trust, and reintegrating into social activities all depend on the support of one’s spouse, family, and friends [20, 21, 32]. Family involvement in the caregiving process and encouraging attitudes increase people’s drive for self-care and reduce feelings of loneliness. Furthermore, interacting with people who have gone through similar things, such as support groups and patient visits, promotes psychological resilience and expedites the normalization process [45, 51]. It is imperative that healthcare providers empower people, fill knowledge gaps, and provide continuous support, especially after discharge [65]. People with stomas can continue their care and adaptation process in a more comprehensive and healthy way when social and professional support are given jointly. These findings demonstrate that managing a stoma is not just a personal challenge but that professional and environmental support networks are essential to the process’s success. For people with stomas, health systems should create organized education and support programs that are educational and psychosocially beneficial.

The research included in this review shows that a network of meanings and interactions formed by the cultural context, in addition to individual coping skills, determines adaptation to living with a stoma. Cultural norms such as bodily integrity, privacy, the perception of “cleanliness/smell,” and visibility can increase the fear of stigma, leading to strategies of concealment, social withdrawal, and reorganization of daily life, according to phenomenological and mixed-methods studies from various countries; on the other hand, family/partner support (sharing care, emotional reassurance, practical assistance) can act as a “buffer” facilitating adaptation [10, 15, 21, 30, 33, 34, 38, 40, 44]. In particular, sexuality and gender roles are noted as culturally sensitive topics; it has been observed that men and women navigate shame, communicate with partners, and the idea of a “reconstructed body” in different ways and that adaptation tactics change according to the situation [32, 47, 51]. Additionally, some research indicates that spirituality/religiosity can be a source that enhances acceptance, hope, and meaning-making; spirituality/religiosity is regarded as a crucial aspect of wellbeing, particularly in long-term survivors [42, 45, 49, 52, 54]. These results imply that stoma care should be supplemented by culturally relevant assessments and interventions, such as family participation, sexuality therapy, spirituality needs, and cultural connotations of stigma and privacy, rather than being restricted to “uniform” training [11, 14, 50, 55].

Research pertaining to the very early and early/midoperative periods (7–15 days–1 year) reveals that people concentrate on issues such as learning self-care techniques related to stomas, managing complications, and addressing uncertainty/body image concerns. When education and home care support are inadequate during this time, adaptation becomes challenging, and quality of life and psychosocial well-being are adversely affected [20, 34, 38, 40, 44]. Even while learning new skills and creating a daily routine might improve adaptation, particularly after two to three months, quality of life and psychological effects may linger if bag/skin issues, social shyness, and stigma perceptions continue [10, 21, 31, 33]. Long-term studies (≥ 2 years, ≥ 5 years, and up to 36 years) indicate that many people learn coping mechanisms and readjust to work/role functioning, but it has been demonstrated that effects in areas such as sexuality, work life, persistent anxieties, and spiritual well-being can fluctuate and not entirely disappear over time [11, 42, 45, 47, 51, 52, 54, 55]. Although most people’s adjustment and quality of life are improved by longer recovery times following surgery, this relationship is not linear, and psychosocial effects may endure over time, depending on complications, social support, and meaning-making processes. Long-term adjustment can be strengthened by a care plan that is staged according to the postsurgical period, with regular quality of life and psychosocial evaluations, peer support, and sexual counselling at later times, and intensive education and emotional support in the early period.

### Strengths and limitations

This meta-synthesis has several limitations. First, there is potential for publication bias, as studies with significant or positive findings are more likely to be published and included in databases. Second, the exclusion of grey literature and non-English publications may have limited the comprehensiveness and cultural diversity of the synthesized evidence. Additionally, the inclusion criteria may have overlooked relevant studies that were inaccessible or not indexed in the searched databases. Third, there was notable heterogeneity in the populations, settings, and study designs of the included studies, which may affect the comparability and synthesis of the findings. Finally, the synthesis was limited in its ability to conduct subgroup analyses by age, gender, or cultural background, which could have revealed important variations in the lived experiences of ostomy patients. In addition, potential sample overlap across some included studies, particularly those involving veteran populations, was considered. To prevent overweight of any specific population, findings were synthesized at a conceptual and thematic level rather than by frequency.

Despite these limitations, this study has several strengths. The fact that the synthesis included 36 studies from fourteen different countries is one of the strengths of this study. Another advantage is that the results of qualitative studies on the experiences of stoma patients have been thoroughly analysed and standardized, and these issues are instructive for academics and clinical practitioners alike. The conceptual consistency attained in terms of the research question focus allowed the findings to be synthesized together, thereby supporting the validity of the meta-synthesis, despite methodological heterogeneity in terms of research design, data collection method, sample, time elapsed after surgery and context.

### Clinical implication

Healthcare providers are encouraged to adopt a holistic and individualized approach when caring for patients with stomas, recognizing the complex interplay of physical and psychological needs such as role adjustments, intimacy, and body image. Multidisciplinary collaboration involving surgeons, stoma therapists, surgical nurses, and mental health professionals can play a key role in ensuring coordinated and long-term care. Surgical nurses may consider taking on advocacy roles, helping to ensure that patient preferences are acknowledged and integrated into decision-making processes. Providing education on technical stoma care, dietary considerations, hygiene, and symptom management starting before surgery and continuing after discharge may support patient adaptations. Educational strategies such as repetitive training, simulation, and visual aids could be useful in reducing anxiety and enhancing confidence. Post-discharge support, including guidance for home care, telephone follow-up, or early identification of complications, may help promote continuity and safety. To address emotional challenges such as social isolation, stigma, or distress, routine mental health screening and access to counselling or peer support may be beneficial. Open discussions related to sexuality and body image could be encouraged, ideally within a culturally sensitive framework that includes both patients and their partners. Support from social workers or occupational therapists may assist with adjustments in daily routines, travel, or return to work. In addition, engagement with peer support groups or online communities might help reduce feelings of isolation. Including family members in the care process may increase patient confidence and independence; therefore, offering emotional and practical caregiving guidance could be considered. Finally, acknowledging spiritual or religious needs, patients potentially through collaboration with chaplains can help individuals maintain their sense of identity and continuity in spiritual practices during their adaptation to life with a stoma.

## Conclusions

This research identified various physical, psychosocial, and care-related barriers that individuals with stomas face in their daily lives. Individuals with stomas often experience strong emotions such as distorted body image, social isolation, shame, loneliness, and anxiety; however, they may tend to develop psychological resilience over time. Their quality of life can be significantly impacted by physical limitations, sleep problems, hygiene issues, and changes in sexual activity. Support from family, spouses, individuals with similar experiences, and healthcare professionals can also help individuals adjust to their stomas and return to their lives. Providing long-term nursing support, information, and counseling services, especially in the post-discharge period, is crucial. For individuals with stomas, this should include individualized education plans, post-discharge nursing staff assistance, and opportunities to connect with others who have experienced similar situations. Future studies should examine the long-term effects of structured support programs focused on helping individuals with stomas return to their normal routines.

## Supplementary Information

Supplementary Material 1.

Supplementary Material 2.

## Data Availability

All the data used in this meta-synthesis were obtained from published studies available in public databases. The full references of all included studies are provided in Table 2.
