# Supplementary material for: Psychosocial effects and quality of life after stoma surgery: systematic review and qualitative meta-synthesis
Source: BMC Psychol. 2026 Jan 23;14:315. doi: 10.1186/s40359-026-03993-w (PMC12964757; doi:10.1186/s40359-026-03993-w)
Supplement: Supplementary file 1 — Supplementary Material 1. [file 40359_2026_3993_MOESM1_ESM.docx]

**Additional file 1** The detailed search strategy conducted in PubMed, Scopus, CIHANL and EBSCO (MEDLINE), and Web of Science (WoS) databases (last search date: December 28, 2024)

| Database | Search | Concept | Key Terms | Search results | Total number of results* |
| --- | --- | --- | --- | --- | --- |
| PubMed | #1 | stoma / ostomy | ( ("stoma"[Title/Abstract] OR "ostomy"[Title/Abstract]  OR "colostomy"[Title/Abstract] OR "ileostomy"[Title/Abstract])  ) | 2.693 | 20 |
|  | #2 | psychosocial | ("psychosocial"[Title/Abstract]  OR "psychological"[Title/Abstract]  OR "social"[Title/Abstract]  OR "body image"[Title/Abstract]  OR "stigma"[Title/Abstract]  OR "psychological well-being"[Title/Abstract]) | 349.139 |  |
|  | #3 | quality of life | ("quality of life"[Title/Abstract]) | 109.962 |  |
|  | #4 | qualitative | ("qualitative research"[Title/Abstract]  OR "qualitative study"[Title/Abstract]  OR "interview*"[Title/Abstract]  OR "phenomenological"[Title/Abstract] OR"ethnographic"[Title/Abstract]) | 206.265 |  |
| Scopus | #1 | stoma / ostomy | ( TITLE-ABS-KEY (“stoma” OR “ostomy” OR “colostomy” OR “ileostomy” ) ) | 51.738 | 156 |
|  | #2 | psychosocial | ( TITLE-ABS-KEY (  “psychosocial” OR “psychological” OR “social”  OR "body image" OR “stigma” OR "psychological well-being"  ) ) | 3.038.596 |  |
|  | #3 | quality of life | ( TITLE-ABS-KEY ( "quality of life" ) ) | 465.320 |  |
|  | #4 | qualitative | ( TITLE-ABS-KEY (  “qualitative” OR “interview” OR “phenomenological” OR “ethnographic”  ) ) | 1.482.040 |  |
| EBSCO  HOST- MEDLINE | #1 | stoma / ostomy | ( XB "Stoma"  OR XB "Ostomy"  OR XB "Colostomy"  OR XB "Ileostomy") | 19 | 8 |
|  | #2 | psychosocial | (XB "Psychosocial Factors"  OR XB "Psychological Factors"  OR XB "Social Factors"  OR XB "Body Image"  OR XB "Stigma"  OR XB "Psychological Well-Being") | 145 |  |
|  | #3 | quality of life | (  XB "Quality of Life"  ) | 539 |  |
|  | #4 | qualitative | (  XB "Qualitative Studies"  OR XB "Interviews"  OR XB "Phenomenology"  OR XB "Ethnography"  ) | 101 |  |
| CINAHL | #1 | stoma / ostomy | ( XB "Stoma"  OR XB "Ostomy"  OR XB "Colostomy"  OR XB "Ileostomy") | 2.417 | 6 |
|  | #2 | psychosocial | (XB "Psychosocial Factors"  OR XB "Psychological Factors"  OR XB "Social Factors"  OR XB "Body Image"  OR XB "Stigma"  OR XB "Psychological Well-Being") | 35.407 |  |
|  | #3 | quality of life | (  XB "Quality of Life"  ) | 91.867 |  |
|  | #4 | qualitative | (  XB "Qualitative Studies"  OR XB "Interviews"  OR XB "Phenomenology"  OR XB "Ethnography"  ) | 133.581 |  |
| Web of Science (WoS) | #1 | stoma / ostomy | TS= (stoma* OR ostom* OR colostom* OR ileostom*) | 178.001 | 169 |
|  | #2 | psychosocial | TS=(psychosocial* OR psycholog* OR social* OR "body image" OR stigma* OR "psychological well-being" OR "psychological wellbeing") | 2.146.221 |  |
|  | #3 | quality of life | TS=("quality of life") | 410.495 |  |
|  | #4 | qualitative | TS=(qualitative* OR interview* OR ethnograph* OR phenomenolog*) | 1.386.112 |  |

*Abbreviations:* * #1 AND #2 AND #3 AND #4; TITLE-ABS-KEY: Title-Abstract-Keywords; XB: Title and Abstract
